# Supplementary material for: Molecular Determinants of Calcitriol Signaling and Sensitivity in Glioma Stem-like Cells
Source: Cancers (Basel). 2023 Oct 31;15(21):5249. doi: 10.3390/cancers15215249 (PMC10648216; doi:10.3390/cancers15215249)
Supplement: Supplementary file 1 [file cancers-15-05249-s001.zip › cancers-2587110-File S1.pdf]

Related to Figure 2

FokI

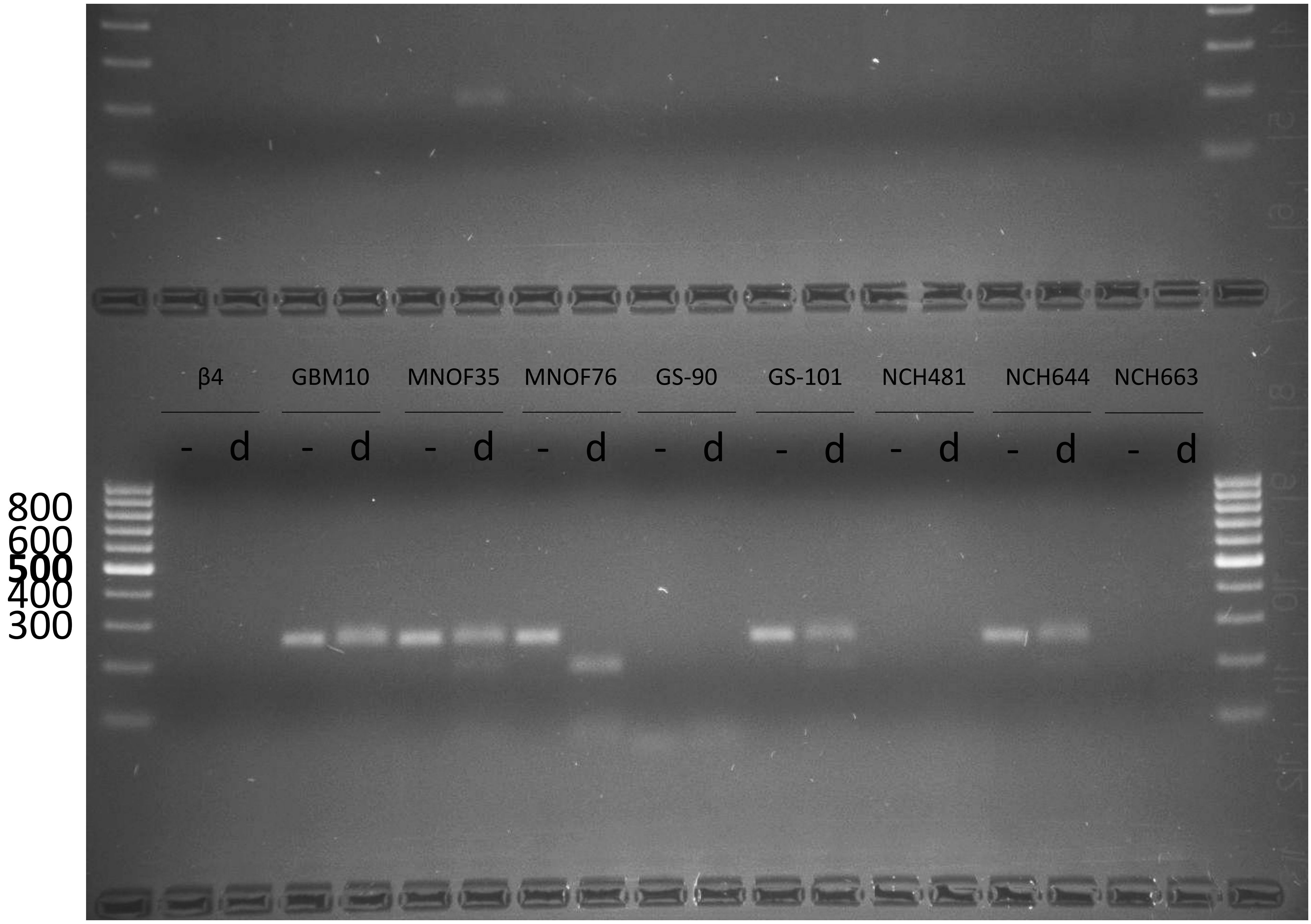

-: undigested; d: digested

Note: A 100 bp ladder was used. For the figure the image was cropped to display GBM10, MNOF35 and MNOF76 representing all VDR polymorphisms

Related to Figure 2

BsmI

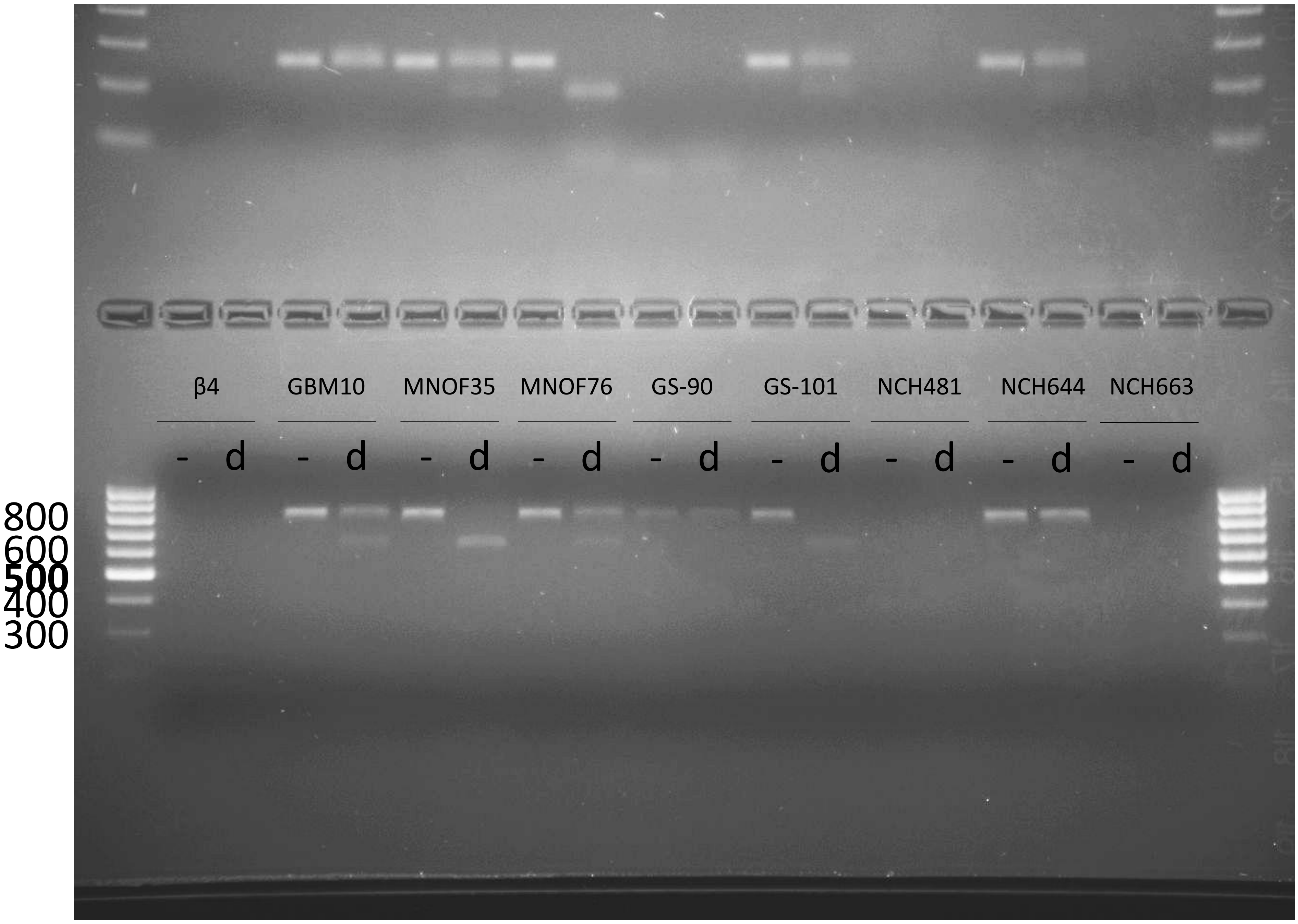

-: undigested; d: digested

Note: A 100 bp ladder was used. For the figure the image was cropped to display GBM10, MNOF35 and MNOF76 representing all VDR polymorphisms

Related to Figure 2

ApaI

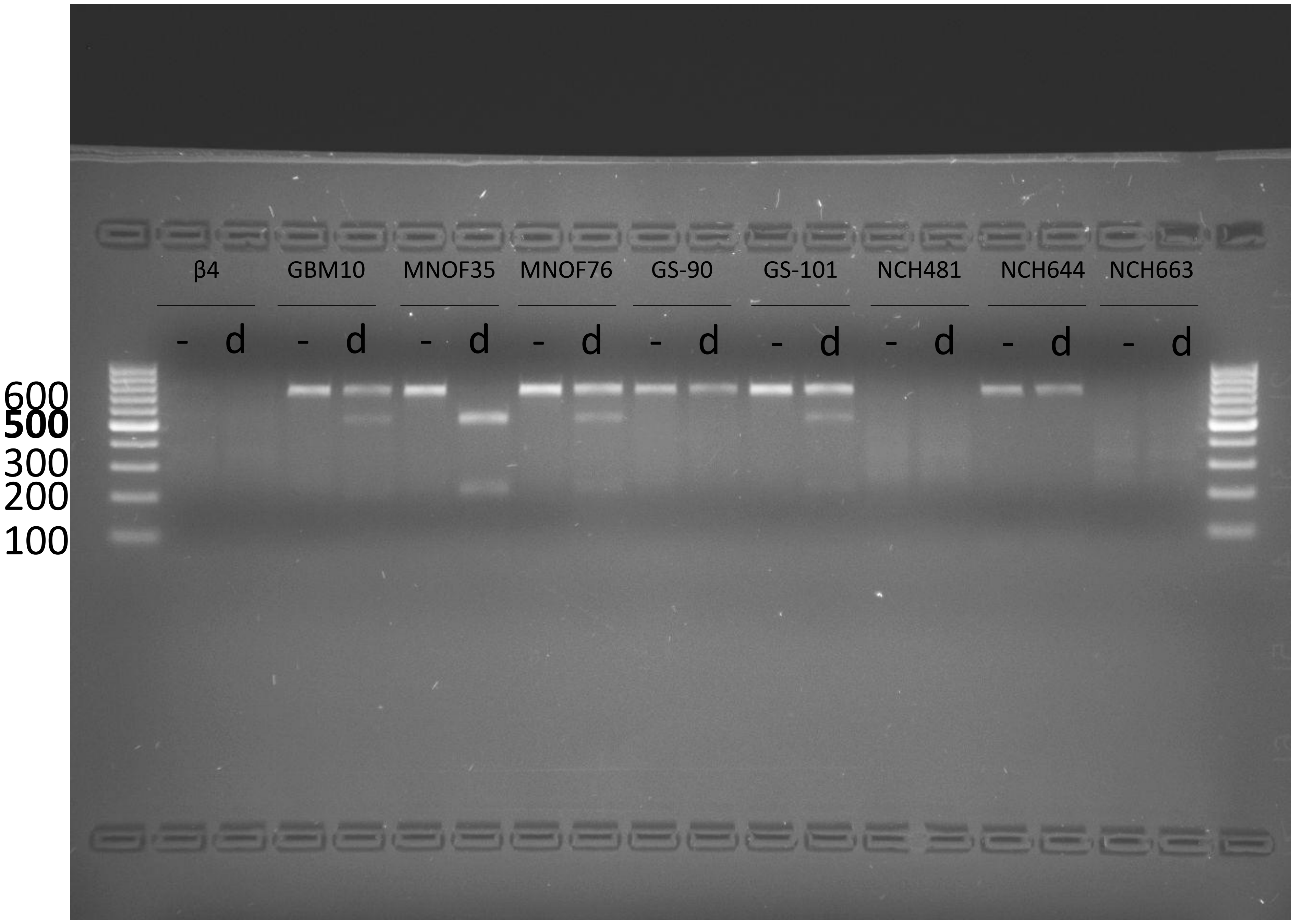

-: undigested; d: digested

Note: A 100 bp ladder was used. For the figure the image was cropped to display GBM10, MNOF35 and MNOF76 representing all VDR polymorphisms

Related to Figure 2

TaqI

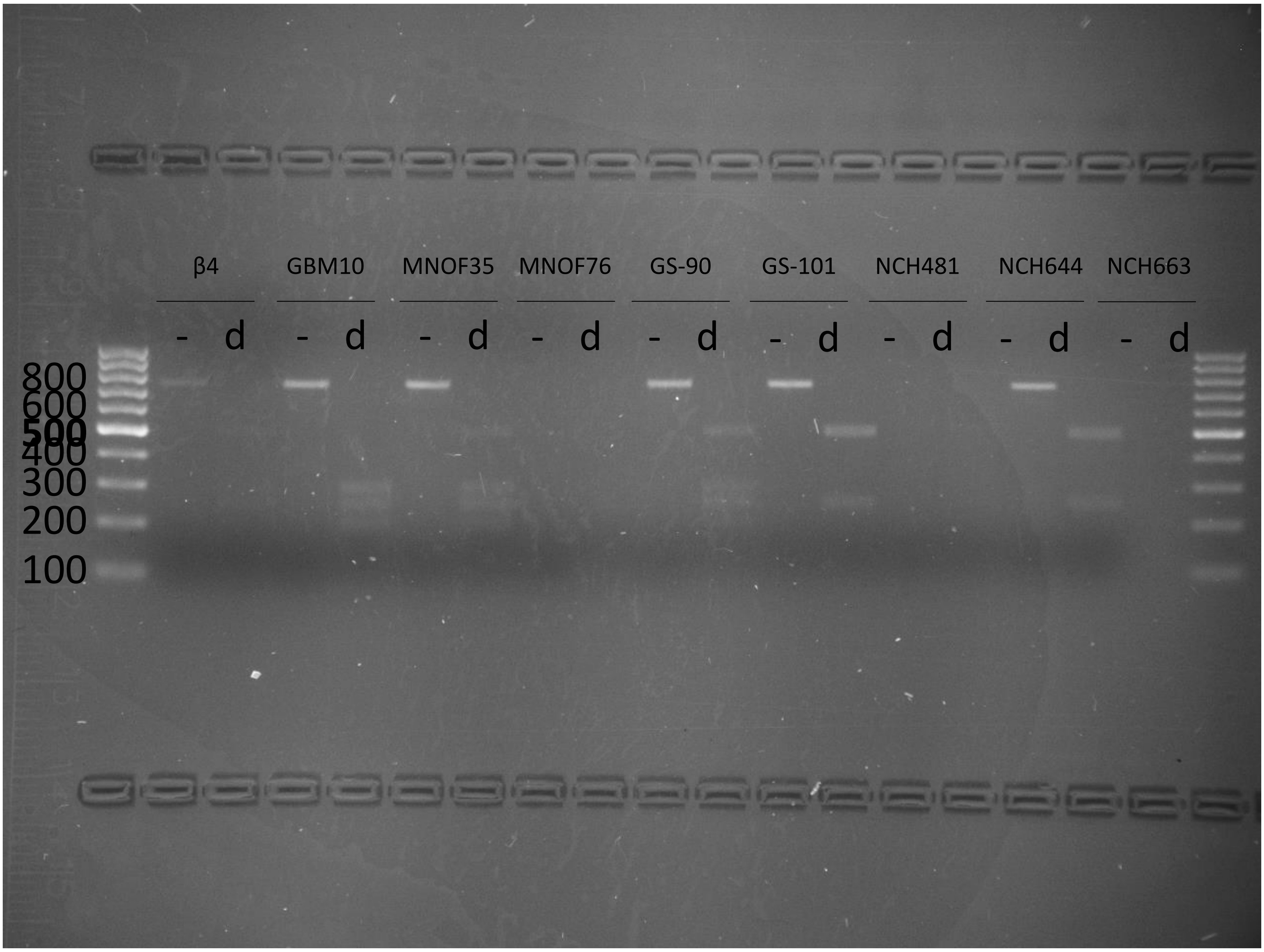

-: undigested; d: digested

Note: A 100 bp ladder was used. For the figure the image was cropped to display GBM10, MNOF35 and MNOF76 representing all VDR polymorphisms
