# Supplementary material for: Molecular Determinants of Calcitriol Signaling and Sensitivity in Glioma Stem-like Cells
Source: Cancers (Basel). 2023 Oct 31;15(21):5249. doi: 10.3390/cancers15215249 (PMC10648216; doi:10.3390/cancers15215249)
Supplement: Supplementary file 1 [file cancers-15-05249-s001.zip › Supplementary Table S1.pdf]

Supplementary Information

*Supplementary Table S1: **Vitamin D receptor allele composition of GSC lines**. For some samples, RFLP analysis did not work in the given time and information is therefore not available (N/A). Alleles are described by capital (homozygote for* mutated *restriction site) or small letters (homozygote for existing restriction site) or both (heterozygote for both).*

**Cell line**	**FokI**	**BsmI**	**ApaI**	**TaqI**	Genotype
high-responders					
β4	*N/A*	*N/A*	*N/A*	*N/A*	*N/A*
GBM10	FF	Bb	Aa	tt	BAt
MNOF35	Ff	bb	aa	*N/A*	baT
MNOF76	ff	Bb	Aa	TT	BAT
GS-90	ff	BB	AA	tt	BAt
GS-101	Ff	bb	Aa	Tt	bAT
NCH481	Ff	bb	aa	TT	baT
NCH644	Ff	BB	AA	Tt	BAt
NCH663	*N/A*	*N/A*	*N/A*	*N/A*	*N/A*
non-responders					
PB1	ff	Bb	AA	TT	bAT
GS-3	Ff	BB	AA	tt	BAt
GS-73	Ff	Bb	Aa	Tt	BAT
GS-80	FF	bb	AA	TT	bAT
HROG52	Ff	Bb	AA	Tt	BAT
HROG63	FF	*N/A*	*N/A*	*N/A*	*N/A*
MNOF107	*N/A*	*N/A*	*N/A*	*N/A*	*N/A*
17/02	FF	bb	aa	TT	baT
